# The role of the pentose phosphate pathway on reproductive functions

**DOI:** 10.1590/1984-3143-AR2024-0130

**Published:** 2025-10-17

**Authors:** José Victor Braga, Janine de Camargo, Mariana Marques, Rafael Gianella Mondadori, Thomaz Lucia

**Affiliations:** 1 Fisiopatologia e Biotécnicas da Reprodução Animal – FIBRA, Faculdade de Veterinária, Universidade Federal de Pelotas – UFPel, Pelotas, RS, Brasil; 2 Centro de Desenvolvimento Tecnológico, Universidade Federal de Pelotas – UFPel, Pelotas, RS, Brasil; 3 Laboratório de Genética e Biotecnologia da Reprodução Animal, Universidade de Passo Fundo – UPF, Passo Fundo, RS, Brasil; 4 Embrapa Suínos e Aves, Concórdia, SC, Brasil; 5 Instituto de Biologia, Universidade Federal de Pelotas – UFPel, Pelotas, RS, Brasil

**Keywords:** metabolism, oocyte maturation, spermatogenesis, embryo development

## Abstract

The application of assisted reproductive techniques (ART) in both farm animals and humans has faced challenges since its inception. Advances in this field have largely depended on a deeper understanding of the metabolic requirements and molecular dynamics of sperm, oocytes, and embryonic development. Glucose, for instance, is commonly utilized as an energy source in *in vitro* procedures. The pentose phosphate pathway (PPP), a pathway parallel to glycolysis, plays a key role in redox regulation via NADPH generation and ribose biosynthesis. This review highlights the role of the PPP in reproductive cells and discusses its potential implications for ART procedures.

## Introduction

Gametes and embryos are highly specialized cells responsible for fertilization and the development of a new, unique organism. Given the limited transcriptional activity in oocytes, spermatozoa, and early embryos (Christou-Kent et al., 2020), they are particularly sensitive to oxidative damage. Consequently, there is a heightened need for a sensitive and rapidly responsive system to maintain redox balance within these cells. Such characteristic is provided by the pentose phosphate pathway (PPP), a metabolic pathway parallel to glycolysis. While the PPP most crucial role lies in redox regulation, it also supports nucleotide synthesis and homeostasis, which are essential for cellular function and health (Stincone et al., 2015).

The glucose-6-phosphate dehydrogenase (G6PD) is the primary enzyme of the PPP and serves as a key source of NADPH and, ultimately, of ribose-5-phosphate (R5P) (Stincone et al., 2015). While both NADPH and NADP are essential for redox maintenance, R5P is crucial for cellular growth (Stanton, 2012). For example, *ΔG6pd* mouse embryonic cells died under induced oxidative stress due to a collapse in the NADP:NADPH ratio, a phenomenon not observed under standard growth conditions (Filosa et al., 2003).

Therefore, cells benefit from the PPP during division, as it provides R5P for nucleic acid synthesis. Additionally, the NADPH produced by the PPP supports cell growth by acting as a reducing agent in the synthesis of fatty acids, cholesterol, hormones, and detoxification reactions, emphasizing the pathway’s importance in cell division and growth (Riganti et al., 2012). For instance, the glutathione system, a primary defense against oxidative stress, relies on NADPH from the PPP for glutathione (GSH) regeneration. Consequently, a weakened PPP impairs NADPH production, compromising GSH regeneration and leading to increased oxidative stress (Riganti et al., 2012). This review focuses on studies highlighting the importance of an active PPP in oocyte maturation, spermatogenesis, spermatozoa capacitation and fertilization, and embryo growth and differentiation. The article emphasizes the connections between the PPP and the structural organization of the oocyte during its maturation, that the sperm differentiation and its capability to fertilize an oocyte are tightly regulated by the PPP and how an embryos’ metabolism and cell proliferation can be similar to cancer cells thanks to this pathway.

## The pentose phosphate pathway (PPP)

**Figure 1:**
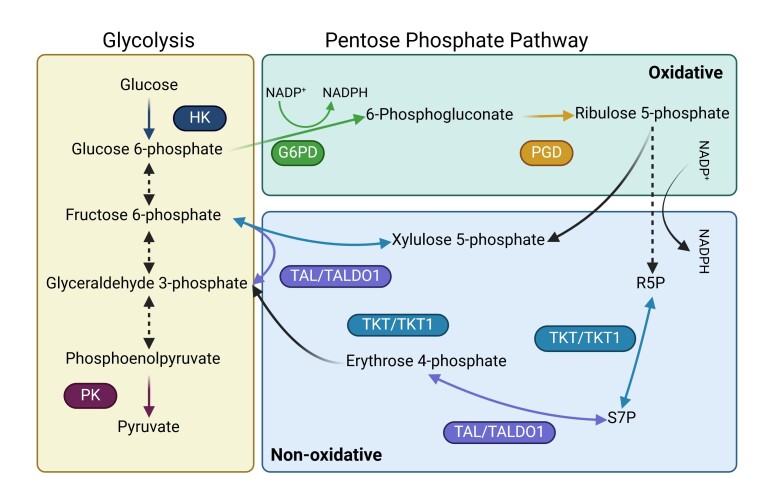
Glycolysis (left panel), oxidative (upper right panel) and non-oxidative (lower right panel) pentose phosphate pathway (PPP) routes, enzymes and products. Arrows and boxes with matching colours represent enzyme activities within the routes. Black and dotted arrows represent direct and stepped conversions in which the enzyme activities are not aborded in this review. Glucose 6-phosphate dehydrogenase (G6PD), hexokinase (HK), pyruvate kinase (PK), 6-phosphogluconate dehydrogenase (PGD), transketolase (TKT) and transaldolase (TAL).

As shown in Figure 1, the PPP is divided into two branches: the oxidative and the non-oxidative (Stincone et al., 2015). The oxidative branch is unidirectional and begins when glucose is converted into glucose-6-phosphate by hexokinase (HK) during glycolysis (Chandel, 2021), ending when 6-phosphogluconate is converted into ribulose 5-phosphate by 6-phosphogluconate dehydrogenase (PGD) (Stincone et al., 2015). Upon entering the non-oxidative branch, ribulose-5-phosphate can be converted to either R5P or xylulose-5-phosphate, which interacts with glycolysis intermediates (Stincone et al., 2015). Throughout both branches, enzymatic reactions facilitate the exchange of NADP for NADPH (Ramos-Martinez, 2017)

The interaction between the PPP and glycolysis becomes more complex in the non-oxidative branch. Transketolase (TKT/TKTL1) and transaldolase (TAL/TALDO1) are the key enzymes operating within this multi-substrate environment. In the non-oxidative PPP, R5P and erythrose-4-phosphate are converted into sedoheptulose-7-phosphate (S7P) – and vice-versa, by TKT and TAL. Both xylulose-5-phosphate and erythrose-4-phosphate serve as substrates for TKT and TAL, producing the glycolytic intermediates fructose-6-phosphate and glyceraldehyde-3-phosphate (Figure 1).

## The PPP on oocyte maturation

*In vivo*, *cumulus-*oocyte complexes (COC) maturation occurs within a dynamic environment where *cumulus oophorus* cells transfer cyclic adenosine monophosphate (cAMP) to the oocyte. This cAMP transfer inactivates the maturation-promoting factor (MPF), inhibiting meiotic progression. Following the luteinizing hormone (LH) surge, cAMP levels decline, leading to MPF activation and the resumption of meiosis.

Simultaneously, the oocyte cytoplasm utilizes pyruvate and other glucose-delivered metabolites provided by the *cumulus oophorus* cells. According to Krisher et al. (2007), both glycolysis and PPP activities are concurrently enhanced during meiosis, resulting in improved cytoplasmatic distribution of organelles and better redox balance through the NADP:NADPH ratio and GSH regeneration in porcine oocytes matured *in vivo*. In contrast, *in vitro* conditions often direct most glucose toward to glycolysis rather than the PPP, leading to improper cytoplasm rearrangement and metabolic misbalance. These factors negatively affect the processes of *in vitro* fertilization and embryonic development.

Mice oocytes treated with stimulators of the PPP exhibited higher rates of germinal-vesicle breakdown, along with increased glucose consumption and lactate production (Downs et al., 1998). It is well established that stimulating PPP leads to the production of glycolysis end-products, given that the non-oxidative branch connects both pathways (Stincone et al., 2015). On the other hand, the addition of either 6-aminonicotinamide (6-AN) – an antagonist of NADP, or dehydroepiandrosterone (DHEA) - which directly inhibits G6PD (Sato et al., 2007) to the maturation media prevented germinal-vesicle break down in mouse COCs, even with HK stimulation via follicle-stimulating hormone (FSH) (Downs et al., 1996). While glucose was consumed, the production of pyruvate and lactate was remarkably reduced. However, removing the oocytes from these inhibitors restored the germinal vesicle breakdown stages, demonstrating the crucial role of PPP activity during oocyte maturation in mice (Downs et al., 1998).

The findings of Downs et al. (1998) raised questions about the activity of PPP in other species. The G6PD activity is regulated by feedback from the NADP:NADPH ratio, as increased levels of NADPH inhibit G6PD activity; while increased levels of NADP stimulate G6PD activity, facilitating NADPH production (Stanton, 2012). In both bovine and porcine oocytes, *in vitro* maturation (IVM) rates decreased when the PPP was inhibited by either NADPH or 6-AN. In contrast, no differences were observed with NADP (Alvarez et al., 2013; Gutnisky et al., 2014) or phenazine ethosulfate (PES) stimulation (Braga et al., 2022), as observed in mice oocytes (Downs et al., 1998). Notably, porcine oocytes treated with brilliant cresyl blue (BCB) showed accelerated MII status (Sato et al., 2007). Although the PPP was both stimulated and inhibited in mouse COCs (Downs et al., 1998), bovine and porcine oocytes appear to be more sensitive to PPP inhibition than to stimulation (Urner and Sakkas, 2005). One possibility is that the *cumulus oophorus* cells could metabolize the stimulators instead of the oocyte, as these cells were not removed prior to IVM.

**Figure 2:**
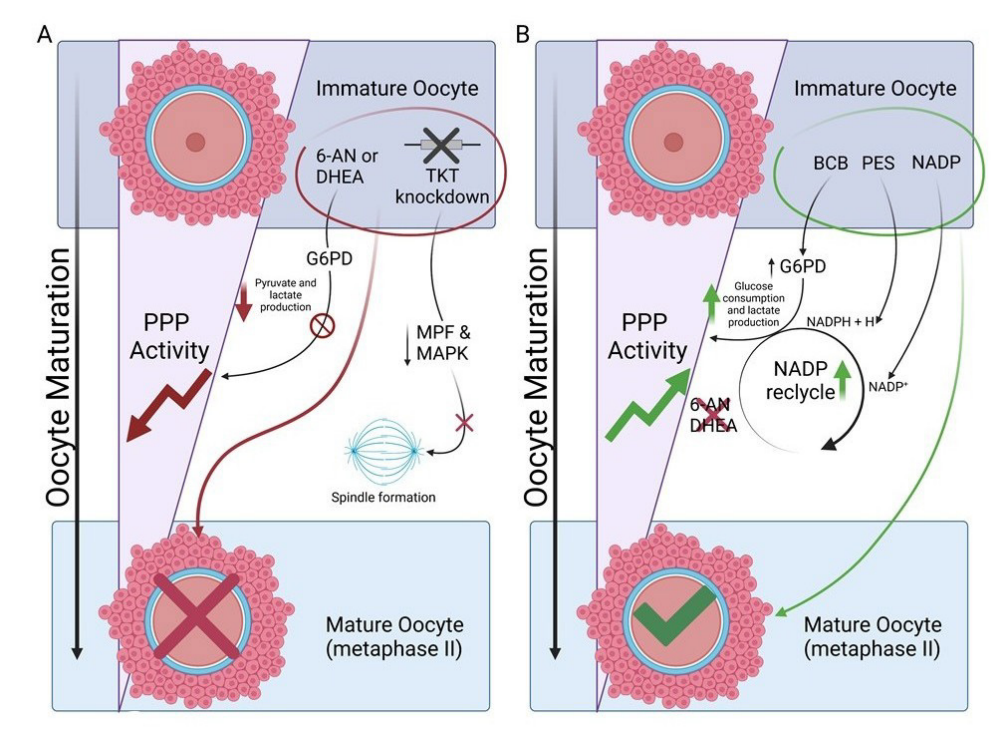
The PPP on oocyte maturation. (A) Immature oocytes cannot reach metaphase II when G6PD activity is blocked and PPP is prematurely reduced after exposure to either 6-AN or DHEA. Also, the knockdown of TKL diminishes MPF and MAPK activities, inhibiting metaphase II by incorrect spindle formation; (B) Immature oocytes can reach metaphase II faster when the PPP is stimulated by BCB or PES, while stimulation with NADP has no effect. 6-aminonicotinamide (6-AN), brilliant cresyl blue (BCB), dehydroepiandrosterone (DHEA), maturation promotion factor (MPF), mitogen-activated protein kinase (MAPK) and phenazine ethosulfate (PES).

Mitochondrial activity and oxidation are necessary for the progression of meiosis during oocyte maturation. Interestingly, the mitochondrial activity of both porcine and bovine oocytes exhibited a similar pattern after PPP stimulation (Alvarez et al., 2013, 2016; Gutnisky et al., 2014). In bovine oocytes, stimulation with NADP reduced oxidative activity and mitochondrial migration, resulting in lower rates of blastocyst formation (Gutnisky et al., 2014). On the other hand, supplementation with NADP did not influence mitochondrial oxidation and migration in porcine oocytes (Alvarez et al., 2013), suggesting that the addition of NADP could not further stimulate PPP activity, since it is already highly active in these oocytes (Alvarez et al., 2016) (Figure 2A).

A hormonal stimulation of rhesus monkeys with either human chorionic gonadotrophin (hCG) or FSH resulted in reduced gene expression of both PGD and TALDO1 in oocytes, compared to those from non-stimulated females (Zheng et al., 2007). While TALDO1 connects the non-oxidative pentose phosphate pathway to glycolysis, PGD is responsible for ribulose-5-phosphate production in the oxidative pathway (Figure 1) (Riganti et al., 2012; Stincone et al., 2015). Additionally, these authors reported that PGD and TALDO1 were predominantly expressed as maternal transcripts, whereas genes involved in glycolysis were only expressed during embryonic development. Nonetheless, flushed and *in vitro* cultured blastocysts presented no difference in PGD and TALDO1 gene expression, indicating that the high PPP activity occurs primarily in oocytes (Zheng et al., 2007). This pattern of PPP activity in the oocytes is consistent with findings in bovine, porcine, and murine studies previously stated (Alvarez et al., 2013; Gutnisky et al., 2014; Jimenez et al., 2013).

Like TALDO1, TKT also serves as a link between PPP and glycolysis. In a study involving TKT knockdown in mice oocytes at the germinal vesicle stage, IVM to the metaphase II (MII) stage was impaired. Although some oocytes did achieve MII, most were arrested in metaphase I (MI) (Kim et al., 2012). The TKT converts S7P to R5P, which serves as a substrate for phosphoribosyl pyrophosphate synthetase 1 and ribokinase. Oocytes with TKT knockdown exhibited reduced expression of these two enzymes, along with defects in spindle structure and decreased activity of both MPF and mitogen-activated protein kinase (MAPK) - key kinases in the maturation process (Kim et al., 2012). The addition of exogenous R5P partially rescued MPF and MAPK activities in TKT knockdown oocytes, promoting MII to a limited extent. This finding is consistent with those of Alvarez et al. (2013, 2016) and Gutnisky et al. (2014), who reported that removing oocytes from PPP inhibitors rescued the maturation of bovine and porcine oocytes (Figure 2B).

As previously discussed, the activity of the PPP within the maturation network is subtle yet essential. While most maturation media utilize glucose as an energy source, successful maturation is only achieved when glucose activates the PPP (Alvarez et al., 2013; Gutnisky et al., 2014). In agreement, the inhibition of the PPP in the studies mentioned here dramatically reduced maturation rates. Conversely, PPP activation with PES can accelerate meiosis from germinal vesicle breakdown to MII in some cases, whereas exposure to NADP shows no relevant effects (Gutnisky et al., 2014). The non-oxidative branch enzymes serve as a metabolic link to glycolysis (Stincone et al., 2015); however, during oocyte maturation, it is also associated with the spindle structure during meiotic progression and provides substrates essential for kinase activities.

In summary, the PPP emerges as a pivotal metabolic route during oocyte maturation, underscoring its indispensable role in reproductive success. The intricate balance between glycolysis and PPP is vital for maintaining metabolic homeostasis, ensuring proper oocyte development and maturation. *In vitro* conditions often fail to replicate the dynamic interplay observed *in vivo*, leading to metabolic imbalances that can adversely affect fertilization and embryonic development. Therefore, optimizing *in vitro* environments to favour PPP activity could significantly enhance oocyte quality and maturation outcomes. Understanding the nuances of PPP's influence on cellular processes provides invaluable insights into improving ART, as the potential to modulate PPP activity holds promise for ground-breaking improvements in reproductive medicine, highlighting the need for continued research and innovation in this critical area.

## The PPP in spermatozoa

For fertilization to occur, sperm capacitation must take place as sperm pass through the female reproductive tract. During this process, the metabolism of sperm cells is primarily directed to fertilization, with ATP generation for flagellar beating taking priority over PPP activity. Thus, both glycolysis and oxidative phosphorylation become the primary metabolic pathways in spermatozoa (Rodríguez-Gil and Bonet, 2016). Nevertheless, the PPP remains essential during spermatogenesis, capacitation, and fertilization (Miraglia et al., 2010; Prokai et al., 2021; Urner and Sakkas, 2005).

### The PPP and spermatogenesis

Metabolic assessments reveal distinct patterns of G6PD activity during spermatogenesis in mice. As spermatogonia grow, multiply, and differentiate, the demand for reduced biosynthesis and building blocks decreases. The transition from mitotic to meiotic divisions corresponds with a gradual reduction in G6PD activity. Particularly in mouse spermatocytes, the G6PD levels are over 20% higher than those found in spermatids and even more elevated compared to spermatozoa, a decline attributed to G6PD nature as a cytoplasmic enzyme (Bajpai et al., 1998).

**Figure 3:**
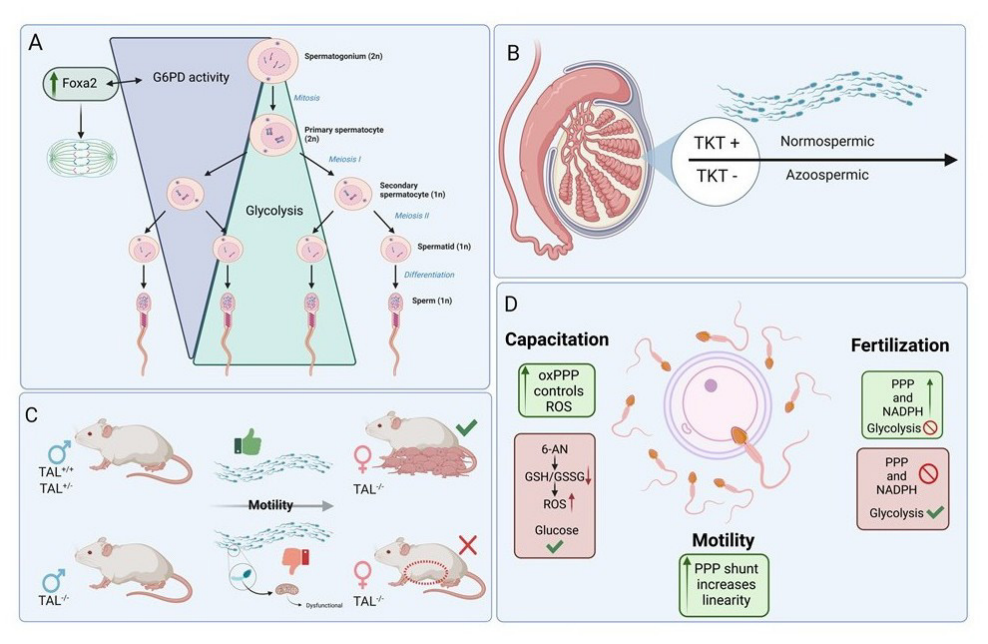
Spermatogenesis, PPP and male fertility. (A) G6PD activity is closely related to Foxa2, which regulates chromatin activity during spermatogenesis. G6PD activity decreases as spermatogonium differs; (B) During spermatogenesis, the absence of TKL in the testis is related to azoospermia in men; (C) Female TAL^-/-^ mice can deliver pups from TAL heterozygotic males, while TAL^-/-^ is related to male infertility due to dysfunctional mitochondria; (D) Spermatozoa capacitation, motility and fertilization capability require an active PPP to control ROS production and to sustain sperm linearity.

Thus, the PPP plays a critical role as germ cells grow and require both NADPH and R5P for biosynthesis. As differentiation progresses, the cytoplasm shrinks, and the activity of both G6PD and PPP declines, redirecting glucose towards glycolysis to generate ATP for flagellar movement (Figure 3A). Prokai et al. (2021) further explored this by examining transcript levels and their connections as undifferentiated spermatogonia began to differentiate into spermatocytes. Using RNA profiling, gene ontology comparisons, and immunolocalization analyses, these authors identified a conserved set of PPP genes upregulated in undifferentiated spermatogonia in rats, mice, and humans (Prokai et al., 2021). Interestingly, the Fork-head Box DNA-binding domain transcription factor 2 (*Foxa2*), which is associated with chromatin regulation and stem cell fate (Cernilogar et al., 2019), was closely linked to both oxidative and non-oxidative PPP activity, as well as cysteine uptake in undifferentiated rat spermatogonia, demonstrating the existence of a tightly regulated redox control during cell growth (Prokai et al., 2021).

The significance of the non-oxidative PPP during spermatogenesis is highlighted by the association between infertility and the absence TKT and TAL, as reported by Rolland et al. (2013) and Perl et al. (2006), respectively. Nearly 1% of men suffer from azoospermia, a condition marked by the absence of spermatozoa in the ejaculate. Immunohistological analyses of reproductive tissues and seminal plasma have distinguished fertile men from those with azoospermia by the presence of the non-oxidative PPP enzyme TKTL1 alongside the glycolytic enzyme phosphoglycerate kinase 2 and L-lactate dehydrogenase C chain (Rolland et al., 2013). These authors defined this combination as a set of germline markers within seminal plasma, with TKTL1 specifically expressed in the testis throughout all stages of spermatozoa differentiation (Rolland et al., 2013) (Figure 3B).

In mice, homozygous deletions of *TKT* and *G6PD (TKL^-/-^* and *G6PD^-/-^*) are lethal (Xu et al., 2002), making so induced *TAL ^-^/^-^* homozygosis a viable model for studying PPP deficiency. This research is significant, as Perl et al. (2006) reported reduced male fertility in heterozygous mice and infertility in homozygous *TAL^-/-^* males, while homozygous females retained normal reproductive function (Figure 3C). The mechanism underlying infertility in *TAL^-/-^* males involved three key factors: abnormal mitochondria and loss of transmembrane potential, which are both essential to sperm motility; accumulation of S7P, indicating impaired conversion of R5P back to glucose 6-phosphate; and the compromised NADPH regeneration, which reduced GSH levels and antioxidant capacity Perl et al. (2006). Therefore, the absence of crucial non-oxidative PPP enzymes disrupts spermatozoa differentiation and compromises motility.

### The PPP and redox control during sperm capacitation

Following differentiation, spermatozoa remain incapable of fertilizing oocytes without undergoing capacitation (Purdy et al., 2022). Capacitation, a series of biochemical and biophysical changes, is crucial for sperm to recognize, bind, and fertilize an oocyte (Purdy et al., 2022). To date, only a few studies in ovine and human models have investigated the role of PPP in spermatozoa capacitation. This highlights a critical need for further research, which could provide valuable insights into the mechanisms of fertilization.

*In vitro* studies by (Miraglia et al., 2010) showed that human spermatozoa exhibit higher oxidative PPP activity and superoxide levels during capacitation compared to non-capacitated spermatozoa. Inhibiting G6PD with DHEA effectively reduced both superoxide levels and sperm capacitation. Although apocynin – a NADPH-oxidase inhibitor - did not reduce the oxidative PPP activity, it lowered NADPH-oxidase activity, impairing reactive oxygen species (ROS) production and sperm capacitation (Miraglia et al., 2010).

To identify a threshold linking ROS levels and sperm capacitation, Donà et al., (2011) categorized human sperm from normozoospermic, fertile men (used as controls) into distinct groups based on ROS production. Their findings showed a ROS-capacitation window where both low and high ROS levels were associated with reduced capacitation and increased number of non-viable cells. Additionally, the use of apocynin impaired tyrosine phosphorylation and increased the number of non-viable cells even in normozoospermic populations. These results indicate a need for balanced ROS generation, highlighting PPP activity as essential for maintaining this balance (Figure 3D).

Western blot and gene expression analyses revealed that both G6PD activity and tyrosine phosphorylation are increased in capacitated ovine spermatozoa compared to those non-capacitated (Luna et al., 2016). Additionally, cAMP administration increased these parameters along with NADPH content and the rate of sperm capacitation, suggesting a link between PPP activity and cAMP (Luna et al., 2016). In ram spermatozoa, inhibiting glycolysis with iodoacetate reduced the motility of cooled sperm - a condition worsened by further inhibition of oxidative PPP by 6-AN (Qiu et al., 2016). The presence of 6-AN also reduced GSH:GSSG ratio and elevated ROS, even when glucose was the primary energy source in the medium. Overall, PPP inhibition proved more damaging to ram spermatozoa than glycolysis inhibition (Qiu et al., 2016) (Figure 3D). Interestingly, spermatozoa from fertile men exhibited increased motility, which was correlated with higher G6PD activity and reduced malondialdehyde levels – a marker for lipid peroxidation (Kurkowska et al., 2020).

Medrano et al. (2005) found that boar sperm stored in glucose concentrations above the optimal range for glycolysis (5 to 10mM) (Marin et al., 2003) could utilize alternative energy sources. The combination of glucose with lactate or citrate helped maintain sperm viability and progressively decreased early acrosome reaction over 168 h of storage. Moreover, Zhu et al. (2022) observed that reducing glucose concentrations in boar sperm extenders enhanced motility, while glucose-free media could not sustain motility.

Furthermore, a subsequent study identified the involvement of the PPP in regulating the linearity of boar spermatozoa (Zhu et al., 2020). This finding contrasts with earlier studies that reported the lack of pentose phosphate shunt activity in boar sperm (Marin et al., 2003), as no carbon labeling was detected in R5P. Zhu et al. (2020) reported that moderate glucose levels in the boar semen extender contributed to itaconate production via the TCA cycle. Itaconate suppresses aldolase A– a glycolytic enzyme that converts fructose 1,6-biphosphate into glyceraldehyde 3-phosphate – and shifts metabolism toward the PPP by interrupting glycolysis. According to other studies, the addition of 6-AN reduced G6PD activity (Alvarez et al., 2013; Downs et al., 1998; Gutnisky et al., 2014) and adversely affected motility kinetics in sperm of rams (Luna et al., 2016; Qiu et al., 2016) and boars (Zhu et al., 2020) (Figure 3D). These findings highlight the metabolic differences in sperm across different species (Rodríguez-Gil and Bonet, 2016).

### The PPP in fertilization

Capacitated and hyperactivated spermatozoa are capable of fertilizing oocytes. Fertilization involves the binding of the capacitated spermatozoa to the oocyte, triggering the acrosomal reaction and enzymatic digestion of the zona pellucida. This process facilitates membrane fusion and the formation of pronuclei (Satouh and Ikawa, 2018). Early studies in sea urchin eggs revealed a rapid activation of G6PD to counteract ROS generated during spermatozoa entry (Shapiro, 1991). In mice, the role of PPP in fertilization has been explored in a series of experiments by (Urner and Sakkas, 1999a, b, 2005). Their findings indicated that glucose metabolism predominantly occurs through glycolysis rather than through PPP during fertilization. However, they found that inhibition using either Cytochalasin B or phloretin before fertilization significantly affected glucose metabolism. Cytochalasin B drastically reduced glycolysis, PPP activity, and sperm penetration rates over time. In contrast, phloretin only inhibited glycolysis, while PPP activity and sperm penetration rates remained comparable to control groups (Urner and Sakkas, 1999a).

In a subsequent study, Urner and Sakkas (1999b) found that spermatozoa-oocyte fusion was impaired in the absence of glucose but could be restored with NADPH supplementation. Thus, the presence of G6PD in the spermatozoa head is required for fusion with oocytes and for maintaining physiological levels of ROS through NADPH oxidation. This process is essential for triggering the resumption of meiosis in fertilized oocytes (Urner and Sakkas, 2005), in agreement with the reported during oocyte maturation (Downs et al., 1998; Kim et al., 2012; Krisher et al., 2007). Those findings suggest that NADPH production via the PPP is required for fertilization and the initiation of embryo development within the oviduct (Figure 3D).

Although glucose was not measured, HK and glucose 6-phosphate were dramatically reduced from the medium to the posterior portion of cows’ oviduct, while G6PD levels decreased from the infundibulum to the entrance of the uterus (Gomes et al., 2010), suggesting a higher PPP activity at the fertilization site. In contrast, Hugentobler et al., (2008) observed no difference in glucose concentration on the entire oviduct or on distinct days of the oestrus cycle. The discrepancies findings between these studies may be attributed to their distinct methodologies. Nonetheless, changes in protein, amino acids, and carbohydrate in oviductal fluid are associated with pregnancy (Rodríguez-Alonso et al., 2020). Furthermore, transferring *in vitro*-produced embryos to oviducts can enhance embryo development, even in trans-species scenarios (Rizos et al., 2010).

The hypothesis that oviductal fluid or its proteins support fertilization and embryonic development *in vitro* is beyond the scope of this article, though it has been investigated (Alcântara-Neto et al., 2022; Hamdi et al., 2018). Treatment of matured oocytes with oviductal fluid prior to *in vitro* fertilization (IVF) did not affect blastocyst rates or morphology; however, it did lead to reduced expression of G6PD and glutathione peroxidase 1 genes on bovine embryos (Cebrian‐Serrano et al., 2013). This reduction is associated with lower levels of ROS damage (Hamdi et al., 2018). A plausible explanation for this observation is that decreased gene expression is not necessarily related to reduced protein or enzyme activity, as gene expression does not always lead to translation (Redel et al., 2012). Therefore, the reduced gene expression observed at that time could indicate an earlier expression, translation, and activation of these enzymes, resulting in diminished ROS levels during the evaluation period.

Summarizing, the PPP plays a multifaceted and indispensable role in various stages of sperm development and function, from spermatogenesis to capacitation and fertilization. During spermatogenesis, the PPP is critical for providing the necessary NADPH and R5P for biosynthesis and redox balance, ensuring proper germ cell maturation. As sperm cells differentiate, there is a strategic shift in metabolism favouring glycolysis and oxidative phosphorylation to meet the energy demands for motility, yet the PPP remains crucial for redox control, particularly during capacitation. Capacitation requires a finely tuned balance of ROS, where the PPP modulates NADPH levels to maintain optimal ROS concentrations, facilitating sperm functionality and fertilizing capacity. During fertilization, the PPP supports essential processes such as the acrosomal reaction and oocyte penetration, underscoring its significance in successful reproduction. The insights gained from understanding PPP dynamics in spermatozoa highlight its potential as a target for therapeutic interventions to improve male fertility and ART outcomes. Future research should focus on elucidating the precise regulatory mechanisms of the PPP in different species and reproductive contexts to enhance our comprehension and manipulation of this critical pathway in fertility treatments.

## The PPP during embryonic development

### The PPP and the Warburg effect during embryo development

Initial embryogenesis relies on the basal oxidation of pyruvate, amino acids, and fatty acids to mitigate oxidative stress until the compaction stage, where glycolysis and the PPP restore balance (Chen et al., 2021). At this stage, the shift of glycolytic intermediates toward the PPP accelerates cell proliferation and redox regulation, albeit with reduced ATP production (Chen et al., 2021). This preference, known as aerobic glycolysis or the Warburg Effect (WE), favours biomass production over ATP and was initially associated with the high proliferative capacity of cancer cells (Warburg, 1956).

Successful embryonic development depends on numerous events. As a totipotent cell, the embryo's potential to multiply and differentiate into all tissues relies on stored mRNA and antioxidants until zygotic genome activation (Zhai et al., 2022), following a precise timeline that demands both energy and biomass synthesis. Although gene expression of G6PD (and thus, oxidative PPP analysis) is well-documented, relative comparisons of the WE in cancer cells versus embryo development have emerged in the last decades (Krisher and Prather, 2012; Redel et al., 2012; Shahzad et al., 2020).

Carbon-radiolabelled studies conducted in the late 80’s and early 90’s were among the first to measure glucose metabolism throughout embryo development (O’fallon and Wright, 1986; Rieger et al., 1992), linking it with energy demands met by pyruvate or lactate after glycolysis (Chandel, 2021). Increased glucose consumption and the release of ^14^CO_2_ from D-[1-^14^C] glucose – processed exclusively in the PPP – from the zygote to the hatched blastocyst were both associated with specific embryonic stages in domestic species and humans (Rieger et al., 1992). Comprehensive reviews on glucose metabolism and the WE are available elsewhere (Krisher and Prather, 2012; Redel et al., 2012).

Long story short, the WE facilitates the accumulation of glycolytic intermediates and their redirection to the PPP during embryonic growth. This process relies on the increased expression of two key WE genes: hexokinase 2; and pyruvate kinase M2. While hexokinase 2 catalyzes the conversion of glucose to glucose 6-phosphate - the substrate for G6PD - pyruvate kinase M2, through a tyrosine phosphorylation site, dramatically slows the conversion of phosphoenolpyruvate to pyruvate, although phosphoglycerate mutase 1 (PGAM1) can do so back-and-forth without ATP exchange. Indirectly, pyruvate dehydrogenase kinase (PDH) is inhibited by phosphate dehydrogenase kinase (PDK), lowering pyruvate accessibility. This reduction in pyruvate availability limits acetyl-CoA input into the TCA cycle, thereby decreasing ATP production while channeling NADPH and ribose through the PPP (Redel et al., 2012). The WE confer a selective growth advantage to proliferating cells. Consequently, alternative energy sources, such as fatty acids or amino acids, must be available to sustain basal TCA cycle activity and ATP production (Krisher and Prather, 2012).

**Figure 4:**
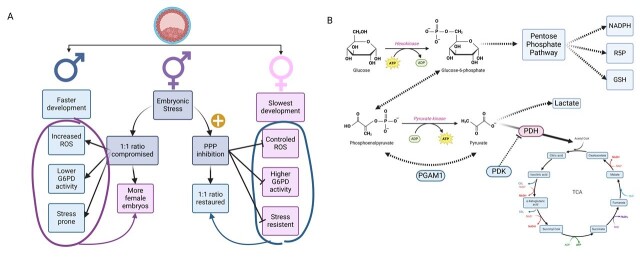
Embryo dimorphism and the Warburg Effect. (A) Female embryos possess more G6PD than males, thus, dimorphism rates are altered during embryonic stress. When PPP is inhibited in stressed embryos, dimorphism rates are restored since female embryos cannot counter-attack ROS production; (B) When pyruvate cannot enter TCA cycle by inhibition of PDH, glucose is shifted towards the PPP and embryonic cells enter a proliferative status in a Warburg Effect manner. Dotted arrows represent Warburg pathways. Pyruvate dehydrogenase kinase (PDH), phosphate dehydrogenase kinase (PDK), phosphoglycerate mutase 1 (PGAM1).

Embryo culture in low-oxygen environments enhances their development, blastomere count, and cryopreservation survival rates (Chelenga et al., 2022; He et al., 2020) while promoting WE characteristics and gene expression. For instance, porcine embryos showed increased expression of TALDO1 and PDK1, genes associated with preventing pyruvate conversion into acetyl-CoA under low oxygen, resulting in a larger inner cell mass and increased total cell number (Redel et al., 2012). Buffalo embryos showed even stronger WE characteristics, with faster development and higher cell number in low-oxygen conditions (Shahzad et al., 2020). These embryos also showed increased expression of glucose transporter 3, phosphofructokinase 1, glyceraldehyde 3-phosphate dehydrogenase, and L-lactate dehydrogenase A, with the later facilitating the conversion of pyruvate to lactate, a hallmark of the WE (Mordhorst et al., 2018) (Figure 4). Additionally, total lipid content also reduced under these physiological oxygen conditions (Shahzad et al., 2020), with similar lipid reductions observed under PES treatment (Barceló-Fimbres et al., 2009; Gajda et al., 2008; De La Torre-Sanchez et al., 2006; Sudano et al., 2011).

Although these studies primarily focused to reduce embryonic lipid content, this objective was largely achieved through stimulation of the PPP. The PES, an electron acceptor, accepts H^+^ from NADPH, regenerating NADP and thereby providing substrate for G6PD. *In vitro*, bovine embryos exposed to PES from the 8-cell stage presented WE characteristics by increasing glucose consumption via the PPP and producing more lactate (De La Torre-Sanchez et al., 2006). These embryos also demonstrated improved survival following freezing-thawing procedures (Sudano et al., 2011), with no observed changes in pregnancy and parturition rates (Barceló-Fimbres et al., 2009). Moreover, porcine embryos showed higher blastomere counts, reduced lipid content and a lower frequency of TUNEL positive cells (Gajda et al., 2008).

Arginine is present in porcine oviducts at higher concentrations than those typically used in culture conditions. When physiological levels of arginine were added to *in vitro* culture, the WE was induced through mammalian target of rapamycin complex 1 (*mTORC1*) stimulation (Redel et al., 2015). The activation of *mTORC1* is a signature of cancer and embryonic cells, promoting glucose uptake (Chen et al., 2017; Herrick et al., 2020; Martin and Sutherland, 2001; Zamfirescu et al., 2021). Through the activity of glutamic pyruvate transaminase 2, glucose is diverted from the TCA cycle, resulting in increased glucose-6-phosphate production via HK1, subsequently resulting in PPP activation (Redel et al., 2015). Interestingly, a decrease in *TALDO1* does not necessarily reflect its protein levels, although neither TKT nor G6PD were analysed.

A seemingly contradictory although intriguing perspective is provided by Cagnone and Sirard (2016), who proposed that overexpression of WE elements could may actually be a response to oxidative stress occurring *in vitro*. In nutrient-enriched culture environments, an excess of nutrients can disrupt embryo quiescence, overstimulate mitochondria, and impair oxidative phosphorylation by increasing ROS production in a Crabtree effect (Cagnone and Sirard, 2016). Therefore, the WE may be established to divert glucose from the TCA cycle and generate NADPH for GSH maintenance. However, embryos cultured with foetal calf serum to reduce ROS content in early stages exhibited compromised development, suggesting that basal ROS levels are required for signalling purposes (Mun et al., 2017), possibly triggering the WE.

The relationship between the PPP and embryonic development with WE characteristics differs from the PPP activity in oocytes, for example. While oocytes utilize G6PD, TKT and TAL activities for redox control, ribose production and spindle regulation during meiosis, embryos with heightened HK activity exhibit WE signatures.

In essence, the PPP and the WE collaborate to support critical metabolic demands during embryonic development. This synergy is pivotal for balancing cellular proliferation and oxidative stress management, as embryos prioritize biomass production over ATP synthesis through aerobic glycolysis. The redirection of glycolytic intermediates to the PPP ensures the generation of NADPH and R5P, essential for biosynthetic and antioxidative processes. This metabolic adaptation highlights the importance of optimizing *in vitro* conditions to mimic the natural embryonic environment, facilitating improved outcomes in assisted reproductive technologies.

### The PPP on embryo dimorphism and implantation

In male mouse embryonic stem cells, deletion of the *G6pd gene* reduced glucose flow into the PPP (Filosa et al., 2003). These cells displayed an impaired capacity to sustain their redox state due to an imbalanced GSH:GSSG ratio, leading to cell death under oxidative stress induced by diamide exposure. In contrast, wild-type cells exhibited increased PPP flux after oxidative stress induction, highlighting the pathway’s role in redox regulation. Interestingly, a 10-fold increase in PPP flux was observed in wild-type cells treated with PES (Filosa et al., 2003). This metabolism control impacts redox status during dimorphic embryo growth. *In vitro,* male embryos grow faster than female embryos due to higher oxidative phosphorylation, albeit with higher ROS generation. Since the *g6pd* gene is X-linked in mammals, its expression in early female blastocysts is nearly double that of males, suggesting greater resistance to oxidative stress (Gutiérrez-Adán et al., 2000). In heat-stressed mouse morulae, fewer male embryos implanted after transfer compared to female embryos, a difference not observed in controls, suggesting that female embryos are better equipped to survive and implant under adverse conditions due to G6PD activity. Furthermore, when heat-stressed morulae were exposed to DHEA, the male-to-female ratio normalized, indicating that inhibiting G6PD hinders female embryo development and attachment (Pérez-Crespo et al., 2005).

Similar to the negative effects observed on mice implantation (Pérez-Crespo et al., 2005), disturbances in the PPP induced by DHEA and 6-AN also equalized interferon-tau (IFN-tau) production between male and female bovine embryos (Kimura et al., 2004). IFN-tau is crucial for embryo attachment to the uterus (Johnson et al., 2023) and this indirect regulation by PPP activity may suggest a higher risk of male embryonic loss, at least in ruminants.

Near implantation, endometrial stem cells undergo decidualization to provide essential factors for embryo development. DHEA disrupts the proliferation and differentiation of both mouse and human endometrial stem cells by reducing the expression of two decidualization markers: prolactin; and insulin-like growth factor binding protein 1 (Frolova et al., 2011). In addition to chemical inhibition, gene expression of both decidualization markers was also reduced following *g6pd* knockdown (Frolova et al., 2011). Defects in decidualization are associated with pregnancy loss.

Thus, the PPP plays a crucial role in regulating redox balance and influencing embryo dimorphism and implantation success. The X-linked nature of the *g6pd* gene confers female embryos with greater resilience against oxidative stress, allowing them better survival and implantation capabilities under adverse conditions compared to male embryos. This is evidenced by the differential expression of *g6pd* and the impact of PPP disturbances on embryo development and implantation, as seen in both murine and bovine models. Moreover, the PPP's influence extends to endometrial decidualization, essential for successful implantation and pregnancy maintenance. Disruptions in PPP activity, whether by chemical inhibitors or genetic knockdown, highlight its potential contribution to reproductive failures, underscoring the pathway's importance in ensuring reproductive success and warranting further investigation.

**Figure 5:**
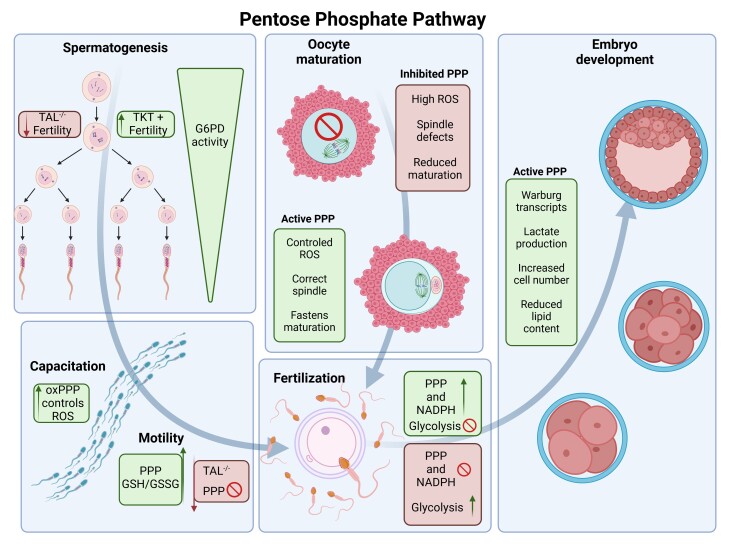
Summary of the pathways of PPP on reproduction. Arrows follow the PPP on the processes of spermatogenesis, oocyte maturation, fertilization and embryo development.

## Conclusion

This review underscores the pivotal role of the PPP in regulating key reproductive processes and highlights its potential as a target for enhancing ART (Figure 5). Our literature review highlights not only the metabolic flexibility conferred by the PPP during oocyte maturation and embryo development but also its critical involvement in maintaining redox homeostasis and facilitating successful implantation. These insights provide a deeper understanding of the metabolic demands of reproduction, offering practical implications for improving ART outcomes. This review highlights the importance of the PPP in gametes and embryonic development, significantly contributing to reproductive metabolism literature. However, further studies on PPP modulation across various species and additional research to address in *vitro* culture limitations are needed to translate these findings into tangible fertility treatment improvements.

## Data Availability

No research data was used.
